# Association of Antibiotic Alterations in Gut Microbiota With Decreased Osseointegration of an Intramedullary Nail in Mice With and Without Osteomyelitis

**DOI:** 10.3389/fendo.2021.774257

**Published:** 2021-12-09

**Authors:** Xingqi Zhao, Zhaohui Zhang, Yiran Wang, Kai Qian, Hanjun Qin, Haoyang Wan, Shihao Wang, Zhengwen Zhu, Siqi Yang, Nan Jiang, Yifang Zhang, Yang Bai, Huimin Deng, Bin Yu

**Affiliations:** ^1^ Department of Orthopedics, Nanfang Hospital, Southern Medical University, Guangzhou, China & Guangdong Provincial Key Laboratory of Bone and Cartilage Regenerative Medicine, Nanfang Hospital, Southern Medical University, Guangzhou, China; ^2^ Department of Gastroenterology, Huizhou Municipal Central Hospital, Huizhou, China; ^3^ Guangdong Provincial Key Laboratory of Gastroenterology, Institute of Gastroenterology of Guangdong Province & Department of Gastroenterology, Nanfang Hospital, Southern Medical University, Guangzhou, China; ^4^ Department of Gastroenterology, The First Affiliated Hospital of Jinan University, Jinan University, Guangzhou, China; ^5^ Editorial Office, Chinese Journal of Orthpopaedic Trauma, Nanfang Hospital, Southern Medical University, Guangzhou, China

**Keywords:** antibiotic, gut microbiota, osseointegration of implant, intramedullary nail, osteomyelitis

## Abstract

Treatment of osteomyelitis requires prolonged antibiotic therapy which significantly alters the gut microbiota. While the influences on bone mass and microstructure have been extensively studied, it is poorly understood what impact the changes in gut microbiota may have on the host response to osseointegration around an intramedullary nail implanted. Here, we explored the influence of gut microbiota on the bone osseointegration process around an implant under two conditions: implantation of an intramedullary nail in the bone marrow cavity and chronic osteomyelitis (CO) induced by *Staphylococcus aureus* infection. Body weight, hepatorenal functions, serum levels of proinflammatory cytokines were monitored. The composition of gut microbiota was assessed *via* 16S rRNA sequencing, and the bone condition was analyzed *via* micro-computed tomography, hematoxylin and eosin staining, Safranin O-fast green and Goldner’s trichrome staining. Osteoblastogenesis and osteoclastogenesis were assessed by detecting tartrate-resistant acid phosphatase and osterix expression. We found that perturbation of gut microbiota (increase in *Proteobacteria* and decrease in *Bacteroidetes*) associated with delayed osseointegration and increased levels of proinflammatory cytokines in the serum (*p*<0.05), lower bone mass (*p*<0.05), deficient endochondral ossification and bone formation, reduced osteoblastogenesis (*p*<0.05) and enhanced osteoclastogenesis (*p*<0.001). Survival rates (*p*=0.002) and bacterial loads (*p*=0.0363) in bone differed significantly between the CO and antibiotic-treated CO mice, but cytokines levels, bone mineral density, and bone formation did not differ, likely because of the severely damaged bone structure. In summary, antibiotic treatment perturbed the gut microbiota and significantly interfered with the bone osseointegration around the nail by increasing proinflammatory cytokine levels in circulation, inhibiting osteoblastogenesis, enhancing osteoclastogenesis, and thus leading to higher pathogen colonization as well as higher mortality postinfection. This report of ours is the first to demonstrate antibiotic-induced alterations in the gut microbiota affect bone osseointegration, helping us understand the role of gut microbiota disorders in osteoblastogenesis and osteoclastogenesis following implant insertion with or without infection.

## Introduction

Bacterial infection is a very serious and common complication of implants which are typically used for patients with fractures or severe osteoarthritis in surgery (1). *Staphylococcus aureus* (*S. aureus*) is the most common pathogen responsible for such bone infections, which, when uncontrolled, can progress to chronic osteomyelitis (CO), a persistent infection in bone caused by pathogenic microorganisms, often resulting in sequestrum formation and bone destruction (2). As a persistent and progressive inflammatory disease, CO remains a huge challenge for clinicians and a burden to patients because of its long duration, complex treatment and high relapse rate (3). Prophylactic antibiotics are commonly prescribed in clinical practice to prevent bone infection. In our previous report, the cure rate for conservative treatment of CO patients was only 26% (4), indicating a necessity for combined antimicrobial and surgical treatments. The clinical guidelines for treatment of *S. aureus*-associated CO generally recommend a combination of antibiotics such as aminoglycosides, gentamicin, and vancomycin (5).

Trillions of microorganisms reside in the human intestinal tract, collectively known as gut microbiota. Changes in gut microbiota associated with antibiotic use are extremely significant, because dysregulation of gut microbiota is associated with numerous abnormalities like inflammatory bowel disease (IBD) (6), obesity (7), non-alcoholic fatty liver disease (8), cardiovascular disease (9) and other conditions. Prolonged antibiotics use perturbs profoundly gut microbiota, which may have adverse effects on musculoskeletal system according to the 2018 International Consensus Meeting on Musculoskeletal Infections (10, 11). One study reported that healthy individuals treated with antibiotics for 1 week or less experienced persistent effects on gut microbiota for more than 6 months, including dramatically decreased microbial diversity and emergence of antibiotic-resistant strains (12). Recent studies have shown that gut microbiota can dysregulate the skeletal metabolism in antibiotic treated animals (13, 14). Cho et al. indicated that slight alterations in gut microbiota early in life were sufficient to interfere with skeletal development and metabolism (13). They administered subtherapeutic antibiotic doses to 4-week-old sex-matched C57BL/6J mice and found that 3 weeks but not 7 weeks of treatment significantly increased bone mineral density (BMD). Another study suggested that the influence of low dose antibiotics on bone mass depended on gender, as long term administration of low dose penicillin to young mice only resulted in increased bone mineral content in female mice (2). More severe dysregulation of gut microbiota may impact the bone more markedly. Hathaway et al. reported that large doses of antibiotic cocktails significantly reduced BMD by enhancing pro-inflammatory response and osteoclastogenesis (14). However, these findings were discrepant possibly because different study designs used.

Until now, only a few studies have investigated the relationship between gut microbiota dysbiosis and bone infection or remodeling. Moreover, no studies have assessed the impact of gut dysbiosis on osseointegration after implant insertion in spite of the fact that rapid and efficient osseointegration between an extraneous implant surface and the host bone tissue is crucial. As microbiota disorders caused by antibiotic use may lead to a series of changes related to bone, we speculate that they may also have impacts on the susceptibility/severity of osteomyelitis and the osseointegration after implantation of an intramedullary nail in the treatment of fracture. Thus, we conducted this study to explore the influence of gut microbiota dysregulation induced by antibiotics on osteomyelitis and osseointegration between an extraneous implant surface and the host bone tissue.

## Material and Methods

### Ethics Statement

All animal experiments of ours were approved by Nanfang Hospital Animal Ethics Committee and conducted in accordance with all relevant ethical principles and guidelines set by the Animal Welfare Act and the NIH Guide for Care and Use of Laboratory Animals.

### Bacterial Strain and Culture Conditions

*S. aureus* strain ATCC 25923 provided by Infectious Diseases Department, Southern Medical University was verified by PCR amplification. *S. aureus* was cultured in tryptic soy broth (BD Biosciences, San Jose, CA, USA) at 37°C in a shaking incubator at 200 rpm overnight for 16 h. Bacteria in the log phase were harvested by centrifugation at 3000 rpm for 10 min, resuspended in sterile phosphate-buffered saline (PBS), and washed 3 times. The *S. aureus* concentration was determined by serial dilution on tryptone soy agar (BD Biosciences) containing 5% sheep blood.

### Animals

Eight-week-old male C57BL/6 mice were used in all experiments. They were originally obtained from Animal Center of Nanfang Hospital and bred and maintained under specific-pathogen-free conditions at an American Association for the Accreditation of Laboratory Animal Care-accredited animal facility at Southern Medical University. They were housed according to the procedures described in the Guide for the Care and Use of Laboratory Animals.

### Experimental Groups

**Figure 1:**
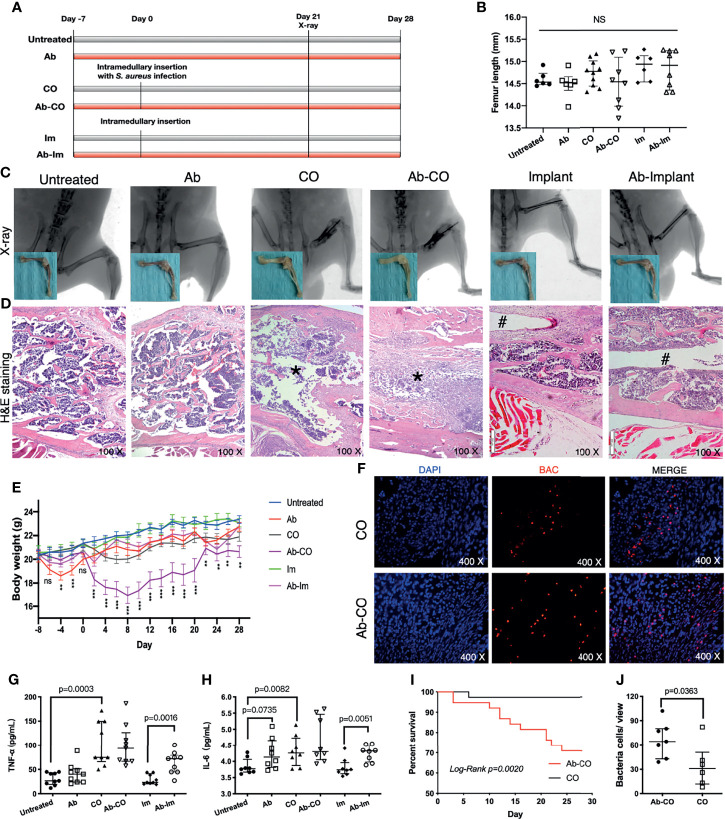
Perturbation of gut microbiota impaired host resistance to chronic osteomyelitis and enhanced proinflammatory response. Specimens were harvested at 4 weeks postsurgery from mice in untreated, antibiotic-treated (Ab), chronic osteomyelitis (CO), antibiotic-treated CO (Ab-CO), implant (Im), and antibiotic-treated implant (Ab-Im) groups. **(A)** Experimental flow chart. **(B)** Length of femur specimen. **(C)** X-ray images of the femur in lateral view and gross photos after soft tissue dissection. **(D)** Histological analysis of the distal femur by H&E staining, osteomyelitis signs (*) in CO and Ab-CO groups, and nail path (#) in Im and Ab-Im groups. **(E)** Body weights of mice in all groups. **(F)** Detection of *S. aureus* in distal femur specimens by FISH. The red signal represents *S. aureus*; the blue signal represents eukaryotic cell nucleus. **(G)** Serum level of TNF-α in all groups (n=9). **(H)** Serum level of IL-6 in all groups (n=9). **(I)** Survival rates for CO and Ab-CO groups (n=38). **(J)** Bacterial cell counts based on FISH results from mice in CO and Ab-CO groups (n=5). Data for body weight, cytokines and bacterial cell counts are shown as the median and interquartile range. Survival data were analyzed using the log-rank test. Body weight data were analyzed by one-way ANOVA. “NS” for not significant, *p < 0.05, **p < 0.01, and ***p < 0.001.

Mice were randomly assigned into 6 groups (**Figure 1A**): (i) untreated group, (ii) antibiotic-treated (Ab) group, (iii) chronic osteomyelitis (CO) model group, (iv) antibiotic-treated CO (Ab-CO) group, (v) implant (Im) group, and (vi) antibiotic-treated implant (Ab-Im) group. Mice in untreated group were reared without any treatment throughout the experiment. Mice in Ab, Ab-CO and Ab-Im groups were fed with drinking water containing an antibiotic cocktail for consecutive 5 weeks, from one week before operation to 4 weeks after surgery (**Figure 1A**). For antibiotic treated mice, an antibiotic cocktail was prepared as previously described (15), containing kanamycin (0.8 mg/mL; Sigma-Aldrich, St. Louis, MO, USA), gentamicin (0.07 mg/mL; Sigma-Aldrich), colistin (0.1135 mg/mL; Sigma-Aldrich), metronidazole (0.43 mg/mL; Sigma-Aldrich) and vancomycin (0.09 mg/mL; Sigma-Aldrich). Kanamycin, gentamicin, and colistin mainly target gram-negative bacteria, vancomycin mainly targets gram-positive bacteria, and metronidazole mainly targets anaerobic bacteria. Mice in Ab-CO, CO, Ab-Im and Im groups suffered from a surgery on day 0 (D0). Detailed experimental procedures for the CO modelling and the intramedullary nail insertion are described below.

### Mouse Surgical Procedures and Infection Model

Southern Medical University Animal Use and Care Committee approved all the procedures of ours. The surgical procedures were modified ones reported by previous work (16, 17). Briefly, mice were anesthetized *via* intraperitoneal injection of tribromoethanol (1.25%). Skin around the right knee joint was prepared and disinfected. After the femoral intercondylar notch was located, a disposable insulin syringe with a 29-gauge needle (KRUUSE, China) was percutaneously inserted into the femoral intramedullary canal. The needle position was confirmed *via* X-ray, followed by manual reaming. The syringe was exchanged for one containing *S. aureus* solution (1×10^7^ colony-forming units [CFU]/mL) (CO model groups) or PBS (implant groups) before 2 µL of bacterial solution or PBS was injected into the intramedullary canal. After a stainless-steel acupuncture needle (length 10 mm, diameter 0.2 mm; Zhongyan Taihe Medical Instrument, Beijing, China) was surgically placed in a retrograde fashion, its tail end was cut off after withdraw by 2 mm. Passive movement of the mouse knee joint was maintained to ensure that the needle had completely entered the intramedullary canal. X-rays were retaken to check the position of the intramedullary nail. Specimens were procured after 4 weeks when all mice were sacrificed.

### Inflammatory Factors and Biochemical Assays

Mouse serum was isolated from the blood by eyeball extirpation and stored at -80°C. TNF-α (CUSABIO, Wuhan, China) and IL-6 (CUSABIO, Wuhan, China) levels were evaluated *via* enzyme-linked immunosorbent assay following the manufacturer’s instructions. Liver function and renal function were tested by Wuhan Servicebio technology.

### Micro-CT Analysis

At week 4 post-surgery, the right femur was obtained from each mouse. After soft tissue around the femur was stripped, the length of femur was measured with a Vernier caliper and fixed in 4% paraformaldehyde for 24 h at room temperature. A high-resolution micro-computed tomography (micro-CT) scanner (SkyScan 1176, Bruker MicroCT, Kontich, Belgium) was used for micro-CT analysis. The scanning procedure voltage was 45 kV with a 555-μA current. The resolution was set to 8.7 μm per pixel. Reconstruction software (NRecon, v1.6.10.1, SkyScan), data analysis software (CTAn, v1.15.4.0, SkyScan), and 3-dimensional (3D) model visualization software (CTVol, v2.2.3.0, SkyScan) were used to analyze the parameters of metaphyseal trabecular bone and diaphyseal cortical bone of the femurs. Cross-sectional images of the distal femur were used for 3D histomorphometric analysis of the trabecular bone and 2-dimensional (2D) histomorphometric analysis of the cortical bone. The region of interest (ROI) in the trabecular bone was drawn starting from 0.3 mm proximally on the distal metaphyseal growth plate and extending proximally for another 1 mm (primary spongiosa; 1.3–3.3 mm as secondary spongiosa). The trabecular bone volume fraction (BV/TV), trabecular thickness (Tb.Th), trabecular number (Tb.N), and trabecular separation (Tb.Sp) were determined from the 3D analysis data and used to represent the trabecular bone parameters. The cortical bone ROI was drawn starting from 4 mm proximally on the distal metaphyseal growth plate and extending proximally for another 0.8 mm. The cortical thickness (Ct.Th), periosteal perimeter (Ps.Pm), and endosteal perimeter (Es.Pm) were determined from the 2D analysis data and used to represent the cortical bone parameters.

### Histomorphological Analysis

Following micro-CT scanning, the femurs were decalcified in 10% ethylenediaminetetraacetic acid (pH 7.4) at room temperature for 4 weeks, dehydrated through a graded ethanol series, and embedded in paraffin. Sections (4 μm) were cut longitudinally and processed for staining with hematoxylin and eosin (H&E), tartrate-resistant acid phosphatase (TRAP), Safranin O-fast green and Goldner’s trichrome. H&E staining (Solarbio, Beijing, China) was performed using standard protocols and visualized under an Olympus BX53 microscope. Standard protocols for Safranin O-fast green staining (Solarbio, Beijing, China) were used to identify collagen fiber and cartilage proteoglycans. Goldner’s trichrome staining (Solarbio, Beijing, China) was used to assess formation of mineralized bone and osteoid according to a standard protocol. TRAP staining (Sigma-Aldrich, St. Louis, MO, USA) was used to detect presence of osteoclasts around the purulent cavity or nail-track following the manufacturer’s protocol. TRAP^+^ mononuclear cells and multinucleated cells containing at least 3 nuclei were identified as preosteoclasts and osteoclasts, respectively, and counted using an Olympus BX53 microscope. For the comparison between untreated group and Ab group, we mainly focus on the bone metabolism of cancellous bone area (primary spongiosa and secondary spongiosa). For the comparison between CO group and Ab-CO group, we mainly focus on the bone microstructure changes around infection focus. For the comparison between Im group and Ab-Im group, we mainly focus on the osseointegration process around intramedullary nail track.

### Immunofluorescence Analyses

For immunofluorescence analysis, sections were blocked with 10% goat serum (Vector Laboratories, Burlingame, CA) at room temperature for 1 h and incubated with primary antibody against osterix (Santa Cruz Biotechnology, Dallas, TX, USA) overnight at 4°C. The secondary antibody, Alexa Fluor 594-conjugated goat anti-rabbit IgG (Proteintech, Wuhan, China), was used to visualize signals, and the sections were subsequently stained with 4’,6-diamidino-2-phenylindole (DAPI; Vector Laboratories) in the dark. Images were acquired using a fluorescence microscope (Olympus BX63). Quantitative analysis was conducted in a blinded fashion using ImageJ software (ImageJ 1.51j8).

### Fluorescence *In Situ* Hybridization (FISH)

In this study, we used the EUB338 probe (5′-GCTGCCTCCCGTAGGAGT-3′) (Biomers, Ulm, Germany) to identify the location and quantify the number of microbes in bone tissue sections (18–20). Briefly, after conventional dewaxing and rehydration of the sections with bone specimen, 0.2 mol/L hydrochloric acid was applied to the tissue for 15 min, followed by 0.5% Triton for 8 min, and washed twice by PBS for 5 min. After washing, the sections were treated with 10mg/mL lysozyme for 15 min. The specimen was naturally dried after PBS washing for 3 times. In the hybridization step, the specimen and probe were covered with hybridization buffer at 37°C for more than 24 hours. After hybridization, the specimen was washed twice with 2×salt-sodium citrate (SSC) (pH 7.5) buffer for 10 min. Cell nucleus were then stained with 1 μg/mL DAPI for 15 min. The slides were observed and analyzed with an epifluorescence microscope (Nikon 80i, Tokyo, Japan).

### Fecal and Intestinal Microbial DNA Extraction and 16S rRNA Gene Sequencing

At the endpoint, all mice were sacrificed to isolate colons. Intestinal samples were collected soon after longitudinal incision of the colon and stored in -80°C before DNA extraction. Microbial DNA was extracted from the intestinal contents samples using a QIAamp DNA Mini Kit (Qiagen, Hilden, Germany) following the manufacturer’s instructions. The V4 region of the 16S rRNA gene was amplified from intestinal contents DNA samples using the primer pair 515F/806R. All libraries were sequenced using the Ion S5™XL platform (Thermo Fisher Scientific, Waltham, MA, USA) by Novogene (Tianjin, China). Sequencing analysis was performed using Quantitative Insights into Microbial Ecology (QIIME, Version 1.9.1) as previous described (21). Briefly, low-quality bases were initially removed using Cutadapt (Version 1.9.1) before paired reads were filtered using the UCHIME algorithm. Clean reads were clustered as operational taxonomic units (OTUs) based on a threshold of 97% identity, and representative sequences were annotated. The Chao diversity index and the number of observed species per sample were used as α-diversity metrics. β-diversity was calculated using unweighted UniFrac distances and represented using principal co-ordinate analysis (PCoA). T-tests were used to analyze differences in observed species, Shanon index, relative abundance and OTU number between Ab-treated and non-Ab treated groups. Raw data for all samples were deposited in the BioProject database at NCBI under BioProject Accession: PRJNA594184.

### Statistical Analysis

GraphPad Prism software was used for all statistical analyses (GraphPad Software Inc.; La Jolla, CA, USA). Data were presented as the median and interquartile range unless otherwise indicated. Survival data were analyzed using the log-rank test while the other data using unpaired t-tests for 2 groups or one-way ANOVA followed by *post hoc* Holm correction for multiple comparisons. Values of *p*<0.05 were considered statistically significant.

## Results

### Perturbation of Gut Microbiota Impaired Host Resistance to CO and Enhanced Pro-Inflammatory Response

We successfully constructed CO and implant models as shown by X-ray and H&E staining of the distal femur (**Figures 1C, D**). Signs of osteomyelitis included swelling of the distal femur and obvious tumefaction of local soft tissue in adjacent regions. In CO and Ab-CO groups, the bone marrow cavities were totally destroyed, with significant abscess formation and sequestrum observed in accordance with pathological features of CO (22). In Ab-Im and Im groups, the nail path was clearly evident.

Mice with gut microbiota dysregulation were more vulnerable to CO as shown by a lower survival rate (**Figure 1I**) in Ab-CO group compared with CO group (*p*=0.002). Simultaneously, *in situ* hybridization assays showed that the number of bacteria in bone was significantly higher (*p*=0.0363) in Ab-CO group than in CO group (**Figures 1F, J**). However, analysis of serum levels of TNF-α and IL-6 in the surviving mice showed no significant differences between the 2 groups (**Figures 1G, H**), possibly because those with worse symptoms died in an early stage so that their clinical symptoms were not detected at the endpoint. Nevertheless, Ab-Im and Im groups differed significantly. Their serum levels of TNF-α (*p*=0.0016) and IL-6 (*p*=0.0051) were significantly enhanced in Ab-Im group compared with those in Im group, suggesting that a systemic inflammatory response was activated following antibiotic treatment in combination with implant insertion. Besides, the significant differences in serum levels of TNF-α (*p*=0.0003) and IL-6 (*p*=0.0082) were also observed between untreated group and CO group, consistent with a previous report (23).

### Antibiotic Dysregulation of Gut Microbiota Reduced BMD

**Figure 2:**
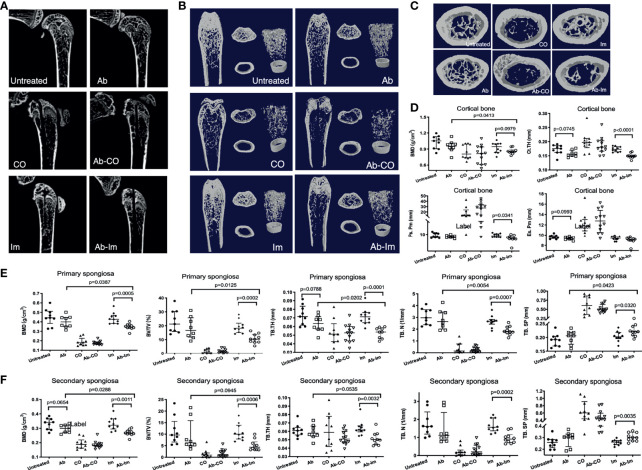
Antibiotic-induced dysregulation of gut microbiota reduced BMD. Distal femoral specimens were harvested and analyzed at 4 weeks postsurgery from mice. Micro-CT analyses were conducted of the trabecular and cortical bone of the distal femur in mice from each group. **(A)** Representative reconstructed 2D sagittal images of the distal femur. **(B)** Representative reconstructed images of the trabecular and cortical bone. Left: coronal plane; Middle: cross-section at the level of the primary spongiosa (upper, extending 0.3–1.3 mm proximally from the distal metaphyseal growth plate) and cortical bone (bottom, extending 4–4.8 mm proximally from the distal metaphyseal growth plate); Right: 3D reconstruction of the trabecular and cortical bone. **(C)** Representative reconstructed cross-sectional images of trabecular bone of the second spongiosa (extending 1.3–3.3 mm proximally from the distal metaphyseal growth plate). **(D)** Characteristics of the cortical bone (n=9) including BMD, cortical thickness (Ct.Th), periosteal perimeter (Ps.Pm), and endosteal perimeter (Es.Pm). **(E)** Characteristics of the primary spongiosa (n=9) including BMD, bone volume fraction (BV/TV), trabecular thickness (Tb.Th), trabecular number (Tb.N), and trabecular separation (Tb.Sp). **(F)** Characteristics of the secondary spongiosa (n=9) including BMD, BV/TV, Tb.Th, Tb.N, and Tb.Sp. All data are shown as the median and interquartile range, analyzed by unpaired *t*-test between untreated and Ab, Ab-CO and CO, or between Ab-Im and Im groups.

Firstly, in the micro-CT analysis to investigate the effects of gut microbiota dysregulation on properties of the cortical and trabecular bones in mice with bone infection or implantation of an intramedullary nail, we could not observe significant results between untreated and Ab groups although some underlying trends concerning Tb.Th in primary spongiosa (*p*=0.0788), BMD in secondary spongiosa (*p*=0.0654) and Ct.Th in cortical bone (*p*=0.0745) suggested that the 5-week broad-spectrum antibiotic cocktail might have exerted influences on bone metabolism. Secondly, the data between Ab-CO and CO groups did not differ significantly (**Figure 2**), likely because prolonged bacterial infection might have induced serious damage to the micro-structures of trabecular bone in these 2 groups, as reflected by the low values of BMD, BV/TV and Tb.N as well as by the high value of Tb.Sp (**Figure 2**). Compared with Im group, mice in Ab-Im group had significantly lower values of BMD, BV/TV and Tb. N and a higher value of Tb.Sp (**Figure 2**), indicating that the properties of trabecular bone in the mice with dysregulated gut microbiota were worse than those in the normal mice following implantation of an intramedullary nail. These results also demonstrated that the gut microbiota might have played an important role in the osseointegration process in normal mice following intramedullary nail implantation. Similar results were observed for the second spongiosa and cortical bone (**Figure 2**).

### Antibiotic-Induced Gut Microbiota Dysregulation Interfered With Osseointegration After Intramedullary Nail Implantation

**Figure 3:**
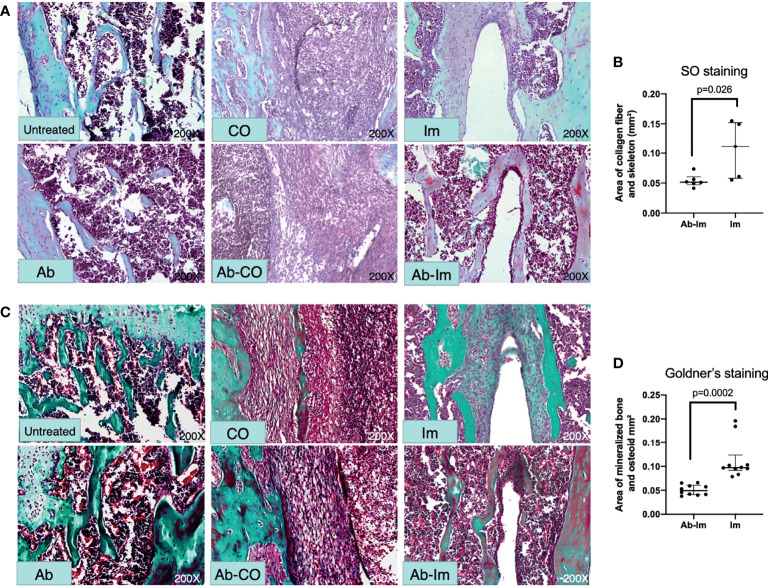
Antibiotic-induced gut microbiota dysregulation interfered with osteoblastogenesis. Distal femur specimens were harvested and stained with Safranin O-fast green (200× magnification) and Goldner’s trichrome (200× magnification) at 4 weeks postsurgery. **(A)** In the Safranin O staining images, deep red indicates cartilage matrix; blue indicates the chondrocyte nuclei; gray-green indicates the cytoplasm, muscle, collagen fiber, and skeleton; red indicates the chondrocyte cytoplasm; and gray-black indicates the cell nuclei. **(B)** Area of collagen fiber and skeleton around intramedullary nail based on Safranin O-fast green staining results from mice in the Im and Ab-Im groups (n=5). **(C)** In the Goldner’s staining images, green indicates mineralized bone; orange-red indicates osteoid tissue; purple indicates cartilage; and blue-gray indicates cell nuclei. **(D)** Area of mineralized bone and osteoid around intramedullary nail based on Goldner’s trichrome staining results from mice in the implant and Ab-implant groups (n=10). Abscess formation and sequestra can be seen in the CO and Ab-CO groups. Mature mineralized bone was evident in the nail path of the Im group but not in the Ab-Im group. Data are shown as the median and interquartile range, and analyzed by unpaired *t*-test.

Considering the influence exerted by gut microbiota on BMD, we further verified whether the gut microbiota affected osseointegration after intramedullary nail implantation. We used Safranin O and Goldner’s staining to visually observe osteoblastogenesis activity. As endochondral bone formation plays a major part in osteoporotic fracture healing (24), we used Safranin O staining to investigate distribution of cartilage and degree of endochondral ossification. Consistently, cartilage was almost absent at the infection site in Ab-CO and CO groups (**Figure 3A**). Ab-Im and Im groups showed mature cartilage around the nail path in different degrees. The cartilage layer was continuous and thicker in Im group but broken and thinner in Ab-Im group (*p*=0.026) (**Figure 3B**). Goldner’s staining further confirmed the level of bone formation (**Figure 3C**). Correspondingly, Ab-CO and CO groups exhibited signs of severe infection with obvious abscess and sequestrum and a very limited number of scattered areas of responsive new bone. New bone and woven bone were observed around the nail path in Im group but not in Ab-Im group (*p*=0.0002) (**Figure 3D**), indicating that antibiotic treatment significantly retarded osseointegration after intramedullary nail implantation.

### Antibiotic-Induced Gut Microbiota Dysregulation Enhanced Osteoclast Activity and Inhibited Osteoblast Function

**Figure 4:**
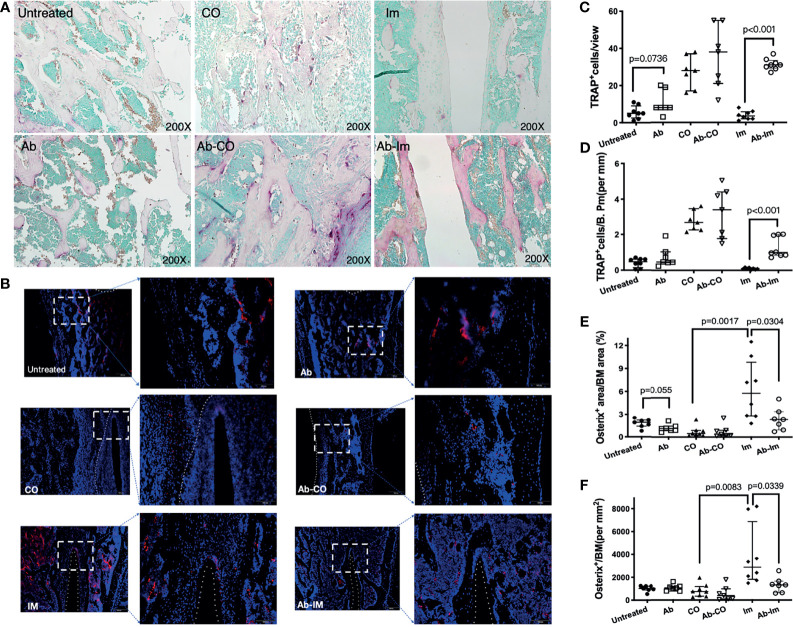
Antibiotic-induced gut microbiota dysregulation enhanced osteoclast activity and inhibited osteoblast function. Distal femur specimens were harvested and stained using TRAP (**A**, 200× magnification) and osterix antibody (**B**, 200× magnification) at 4 weeks postsurgery. Osteoclasts expressing TRAP are shown in purplish red, demonstrating osteoclastogenesis activity. Osterix expression in the femoral specimens was highlighted by an immunofluorescence assay and demonstrated osteoblastogenic activity. Nuclei are stained with DAPI. **(C)** TRAP^+^ cell counts in view. **(D)** TRAP^+^ cell counts in trabecular bone. **(E)** Osterix^+^ cell counts in bone marrow cavity. **(F)** The area percentage of osterix-positive signals. Cell counts and area percentages are shown as the median and interquartile range. All data are analyzed by unpaired *t*-test between untreated and Ab, Ab-CO and CO, or between Ab-Im and Im groups.

TRAP and osterix staining were performed to further understand the influence of gut microbiota on activities of osteoclasts and osteoblasts [TRAP is a widely used marker of osteoclasts (22)]. Quantification results showed that many TRAP^+^ osteoclasts were observed in the bone marrow cavities (**Figures 4A, C, D**) and the mice in Ab group had more TRAP^+^ cells in bone than in untreated group, but the difference was not statistically significant, only exhibiting a clear tendency consistent with the findings by Micro-CT. TRAP^+^ cell frequency did not differ statistically between Ab-CO and CO groups either, though the Ab-CO mice had higher average numbers of TRAP^+^ osteoclasts than did the CO mice. Notably, significantly more TRAP^+^ osteoclasts were found in Ab-Im group than in Im group (**Figures 4A, C, D**), indicating that antibiotic perturbation of the gut microbiota might have recruited osteoclasts to the host bone tissue contacting the extraneous implant surface. Osterix immunofluorescence assays (**Figure 4B**) showed significantly reduced osterix expression in Ab-Im group (**Figures 4E, F**), also indicating that gut microbiota dysregulation inhibited osteoblastogenesis as osterix is an osteoblast-specific transcription factor mainly responsible for osteoblastogenesis (25).

### High-Dose Antibiotic Treatment Induced Significant Gut Microbiota Dysregulation in Mice

**Figure 5:**
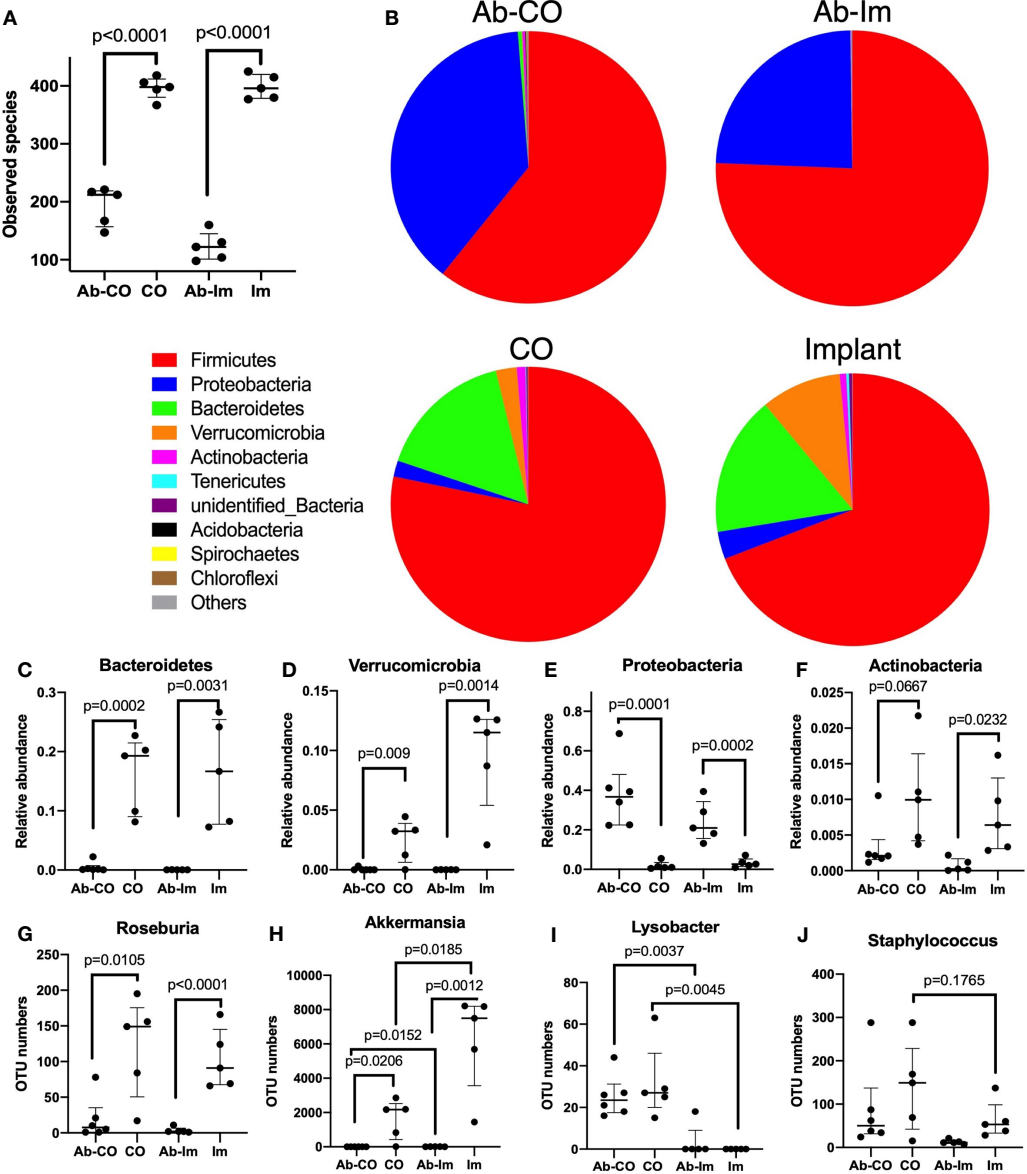
A high-dose broad-spectrum antibiotic cocktail significantly disrupted the host gut microbiota. After 5 weeks of antibiotic treatment, intestinal contents specimens were harvested and analyzed *via* 16S rRNA sequencing from mice in untreated, antibiotic-treated (Ab), chronic osteomyelitis (CO), antibiotic-treated CO (Ab-CO), implant (Im), and antibiotic-treated implant (Ab-Im) groups (*n*=5–6 mice/group). **(A)** Observed numbers of species for all groups; **(B)** The relative composition of bacterial phyla shown in all groups. **(C–F)** Relative abundance of *Bacteroidetes*, *Verrucomicrobia*, *Proteobacteria*, and *Actinobacteria* phylum. **(G–J)** OTU number of *Roseburia, Akkermansia, Lysobacter*, and *Staphylococcus* genus. Data for observed species, OTU number and relative abundance are shown as median and interquartile range. All data were analyzed by unpaired *t*-test between Ab-CO and CO, or between Ab-Im and Im groups.

Dysregulation due to gut microbiome destruction was confirmed by 16S rRNA sequencing (**Figure 5** and **Supplementary Figure 3**). Results of PCoA analysis (**Supplementary Figure 3D**) provided information that dots in intra-group samples assembled while inter-group samples dispersed obviously, showing that current intestinal content samples were qualified and acceptable. Accordingly, the distance between Ab-treated groups and non-Ab-treated groups was definitely far, further proving that the discrepancy in microbiota composition between those groups was due to the antibiotic treatment (**Suplementary Figure 3**).

As expected, the amount and composition of gut microbiota were significantly perturbed in the Ab-treated mice, as shown by a much lower number of species observed (**Figure 5A**) as well as by the absence of specific microbes compared with the non-Ab-treated control mice. In Ab-CO and Ab-Im groups, *Bacteroidetes* (**Figure 5C**) and *Verrucomicrobia* (**Figure 5D**) phyla almost disappeared, indicating that broad-spectrum antibiotic mixtures disrupted the gut microbiota composition seriously. Consistent with a previous report, chronic antibiotic treatment induced a gut microbiota population characterized by obviously increased relative abundance of *Proteobacteria* (**Figure 5E**) phylum (26). The proportion of *Proteobacteria* phylum increased dramatically in Ab-treated groups, partially because some microbes belonging to *Firmicutes* phylum might have been eliminated. For example, OTU number of *Roseburia*, a genus belonging to *Firmicutes* phylum, mainly responsible for short chain fatty acids (SCFAs) production, was significantly decreased (**Figure 5G**) in both Ab-CO and Ab-Im groups. Moreover, OTU numbers of multiple recognized beneficial or commensal bacteria, like *Akkermansia* genus (**Figure 5H**) from *Verrucomicrobia* phylum and *Bifidobacterium* genus (**Suplementary Figure 3C**) from *Actinobacteria* phylum, were also decreased dramatically after consecutive use of antibiotics.

In addition, we also discovered significant results by comparison between CO and Im groups. OUT numbers of *Lysobacter* (**Figure 5I**) and *Staphylococcus* (**Figure 5J**) were higher and that of *Akkermansia* (**Figure 5H**) was significantly lower in the mice with osteomyelitis than in the mice without osteomyelitis but only with an intramedullary nail (**Figure 5**).

## Discussion

Recent research has focused on potential associations between gut microbiota and bone. Here we explored the role of antibiotic-induced perturbation of gut microbiota in osseointegration in response to an intramedullary nail in mice with or without osteomyelitis. Our results demonstrated that perturbing gut microbiota by antibiotics associated with inefficient osseointegration around an intramedullary nail, increased pro-inflammatory cytokine levels in serum, lower bone mass, deficient bone formation, inhibited osteoblastogenesis and enhanced osteoclastogenesis. Survival and bacterial load differed significantly between mice in CO and Ab-CO groups, but their serum cytokine levels, BMD, or bone formation did not differ probably because of severe damage to bone structure. Accordingly, these findings indicate that the disturbed gut microbiota might have been probably associated with the antibiotic mediating passive resistance to osteomyelitis and insufficient osseointegration in mice.

In current study, we disrupted the gut microbiota in 8-week male C57BL/6 mice by 5-week-treatment with drinking water containing high-dose broad-spectrum antibiotics. The 16S rRNA analysis showed that consecutive antibiotic treatment led to significantly fewer species observed, together with almost disappearing of relative abundance of *Bacteroidetes* and *Verrucomicrobia* phyla and correspondingly an enriching proportion of *Proteobacteria* phylum. The expansion of *Proteobacteria* is commonly observed in the hosts with dysbiosis, and typically associated with sustained intestinal inflammation (27) and gut barrier dysfunction (28), and has been reported in correlation with impaired bone mechanical properties in mice (26). *Akkermansia muciniphila* (*A. muciniphila*), a major member from *Verrucomicrobia* phyla, which was almost absent after this antibiotic treatment (**Supplementary Figure 3E**). *A. muciniphila* has been recently regarded as a promising candidate for next-generation probiotics thanks to its beneficial characteristics, like producing SCFAs (29), inhibiting inflammatory response through expanding regulatory T (Treg) cells (30), and sustaining gut barrier function (31). The wholesome gut microbiota net plays a critical role in sustaining diverse physiological functions in host, and thus impairing *Bacteroidetes* and *Verrucomicrobia* as well as other gut microbes in Ab-treated mice may inevitably lead to shortage of nutrition and SCFAs, uncontrolled inflammation and other chain effects.

According to the result of microbial analysis, the increased OTU number of *Staphylococcus* was observed in osteomyelitis model mice, and this was probably associated with exogenous injection of *S. aureus*. In addition, the increased abundance of *Lysobacter* in CO groups (**Figure 5I**) was possibly an adaptive enrichment since a couple of *Lysobacter* species have been reported with potent antimicrobial abilities to inhibit methicillin-resistant *S. aureus* (MRSA) by releasing metabolites (32). Furthermore, a significantly increased number of *A. muciniphila* was observed in the mice after implantation of an intramedullary nail (**Figure 5H**), and a newly study has demonstrated the role of *A. muciniphila* in mediating protection from osteoporosis through exerting directly regulation in bone by secreting extracellular vesicles (33).

Numerous researches have explored the mechanisms of gut-bone axis. The possible mechanisms for the finding of the present study should be also explored that perturbation of gut microbiota mediating restricted osseointegration. First of all, malnutrition and hepatorenal damages caused by long term and high dose antibiotic treatment should not be ignored. Calcium, which is considered to have positive effects on bone mass throughout life, should be affected under a long-term use of antibiotics since gut microbiota metabolites are reported to have great impacts on calcium absorption in the intestine (34). In the present study, although there was no significant difference in femur length (**Figure 1B**), hepatorenal function or structure (**Supplementary Figure 1**) between Ab-treated and non-Ab-treated mice, the Ab-treated mice did experience obvious weight loss throughout the experiment (**Figure 1E**), indicating that malnutrition induced by antibiotics may probably exert an influence on deficiency in bone remodeling.

Apart from nutritional factors, the mechanisms for antibiotic induced impaired resistance to infection and deficient osseointegration around an implant in the present study might probably be direct regulation by bacterial metabolites and indirect regulation by immune system. Numerous studies have discussed the mechanisms for gut microbiota metabolites mediating bone metabolism. SCFAs, which have a versatile role in human metabolism (35), are major products by gut microbes, mainly including formate, acteate, propionate and butyrate. Study has demonstrated that SCFAs directly support bone remodeling and suppress bone resorption by inhibiting osteoclast activities *via* activation of free fatty acid receptor 2 (FFAR2), the natural ligands for SCFAs found on a wide range of cell types (36). Moreover, they affect bone metabolism by regulating endocrine as well. Insulin-like growth factor (IGF-1), crucial to bone health and growth during the postnatal period (37), was recently recognized as a connector between the gut and bone (38, 39). Yan et al. discovered that orally supplementing SCFAs to antibiotic-treated mice made up for the loss of serum IGF-1 induced by antibiotics, thereby improving bone metabolic abnormalities in mice (38). Hydrogen sulfide (H_2_S), another sort of bone-regulating molecules, stimulates bone formation and postnatal skeletal development (40). Intestinal H_2_S is produced by gastrointestinal cells and by gut microbes (41), accounting for modifying gut microbiota composition and maintaining body health. The serum H_2_S level was found significantly lower in ovariectomy mice, a common model of postmenopausal osteoporosis, and oral treatment with H_2_S-donor GYY4137 to ovariectomy mice significantly promoted osteoblastogenesis through activating Wnt signaling by production of Wnt ligands in the bone marrow (42). In our study, production of metabolites like SCFAs was dampened greatly in Ab-treated mice, since most of SCFAs-producing microbes, like *Akkermansia, Bifidobacterium* and *Roseburia* were significantly inhibited after antibiotic treatment (**Figure 5**, **Supplementary Figure 3C**). Therefore, it may be a possible mechanism that gut dysbiosis results in shortage of bacterial metabolites, thereby leading to deficiency in bone formation and facilitating bone resorption during osseointegration.

Another important mechanism may be explained by immunoregulatory effects. The regulation of skeletal development and homeostasis by interactions between immune cells and bone cells is extensively investigated by osteoimmunology researches (43). More like a bridge, the immune system has versatile impacts on bone metabolism. Simply, an inflammatory state promotes bone resorption while an anti-inflammatory state promotes bone formation. Gut microbiota may regulate bone metabolism indirectly by influencing local and systemic immune systems. As is known, commensal bacteria establish their homeostasis with host *via* inducing expansion of Treg cells (44), which are a strong driving force for bone formation as well. Treg cells inhibit osteoclastogenesis basically *via* direct cell-cell contact or producing various cytokines, including TGF-β, IL-4, and IL-10 (45). Bacterial metabolites regulate skeletal metabolism not only directly but also by inducing anti-inflammatory response. Recent study showed that 4-week treatment with antibiotic mixture significantly decreased the concentration of butyrate by about 50% and inhibited the expansion of Treg cells in both gut and bone marrow in mice (46, 47). Butyrate is regarded as a necessity for restoring parathyroid hormone-induced anabolic action in bone (46), because it is capable of promoting bone formation by triggering Wnt10b release and activating Wnt signaling in osteoblastic cells through inducing differentiation of Treg cells *via* GPR43-independent signaling (47, 48).

Gut microbiota disorder is closely correlated with inflammatory state and accounts usually for pathogenesis of IBD, manifesting an increase in serum levels of TNF-α and IL-6 (49). Crohn’s disease, a kind of IBD with specific gut microbiota disorder, is usually in association with osteoporosis. It is induced not only by the glucocorticoid therapy but also by increased pro-inflammatory cytokines including IL-6 and TNF-α (50). These pro-inflammatory cytokines are negative regulators of bone formation, inhibiting osteoblast differentiation (51) and stimulating osteoclastogenesis (52, 53). Our present finding that the serum levels of TNF-α and IL-6 were significantly enhanced in Ab-Im group compared with Im group may partially explain the impaired osseointegration and osteoblastogenesis activity observed. This finding is consistent with that of Guss et al. that perturbation of gut microbiota significantly increased the circulating level of TNF-α and dysregulated osteoimmune cross-talk, thereby driving pro-osteoclastic effects (26).

The balance between bone formation and bone resorption is deeply affected by the condition of gut microbiota; the activities of osteoblastogenesis and osteoclastogenesis determine the outcomes of bone remodeling and osseointegration. TNF-α may recruit osteoclast precursors in bone marrow, playing a critical role in activating pathogenic osteoclastogenesis and promoting bone resorption (54). Consistently, our present experiments have indicated that inflammatory state induced by perturbed gut microbiota may significantly block the implant osseointegration process after we observed higher serum levels of TNF-α and IL-6, a lower bone mass, insufficient formation of mature cartilage, woven bone and new bone, more TRAP^+^ cells and less osterix^+^ cells in Ab-Im group than in Im group. The potential mechanisms for this indication may probably involve lack of nutrition, shortage of SCFAs and inflammatory state mediated by antibiotic perturbation of gut microbiota.

In addition, the state of gut microbiome also influences host resistance to osteomyelitis. All the mice in our Ab-CO and CO groups suffered a surgery and exogenic infection, but only those in Ab-CO group showed a much higher death rate and a much higher bacterial load in bone. This result is consistent with that in a previous study by Hernandez et al. (55). They disrupted the mice gut microbiota by antibiotic treatment for an 8-week period prior to surgery and found that the incidence of periprosthetic joint infection was significantly increased in mice with gut dysbiosis.

Our current data stress the importance of an intact and wholesome net of gut bacteria for a host in the condition of bone infection or osseointegration after intramedullary nailing. However, the limitations of the present study must be considered when interpreting its findings. First, since we perturbed the gut microbiota in mice of 8-week old when they were still growing, the possibility should not be ignored that our findings might have been influenced by the skeletal development before 16-week of age in the host. Secondly, our experiments were conducted only in male mice and female ones would have possibly led to different responses. Thirdly, long term use of antibiotics not only perturbs gut bacteria but also possibly promotes fungal infection in gut (56, 57). Thus the possible influence from fungal infection following antibiotic treatment on osseointegration should not be ignored as well. Fourthly, it was almost impossible for us to find out the exact mechanisms for specific microbes by use of broad-spectrum antibiotic mixture in the present study, since different bacteria exert entirely different functions and should possibly play different roles in bone metabolism. Further researches targeting specific microbe or specific pathway are needed to investigate the mechanisms of gut microbiota for mediating osseointegration. Nevertheless, our primary concern here was to investigate whether or not gut microbiota disorder might exert influences on the host response to intramedullary nailing and osteomyelitis.

In conclusion, all our findings have pointed out that the gut microbiome was associated with the host resistance to osteomyelitis and the implant osseointegration process. We have also tried to explained the possible mechanisms for this association, but more powerful evidence is to be provided by future researches. The present study of ours is only a tentative attempt to explore the effects of changes in gut microbiota on implant osseointegration and osteomyelitis. Although there is much to be clarified, our current data substantially advance our knowledge of the role of gut microbiota dysregulation in osteoblastogenesis and osteoclastogenesis in response to intramedullary nailing and osteomyelitis. Further studies are needed to confirm the deterioration of skeletal metabolism following antibiotic-induced dysbiosis of gut microbiota under different conditions and to clarify the exact molecular mechanisms involved.

## Data Availability Statement

The datasets presented in this study can be found in online repositories. The names of the repository/repositories and accession number(s) can be found in the article/**Supplementary Material**.

## Ethics Statement

The animal study was reviewed and approved by Nanfang Hospital Animal Ethics Committee.

## Author Contributions

XZ and ZHZ performed the experiments with bacteria and mice, analyzed data and contributed to manuscript revision. YW analyzed the data and contributed to manuscript revision. KQ, HQ, HW, SW, ZWZ, and SY performed the experiments with mice. NJ contributed to manuscript revision. YZ contributed major and careful revisions in language and ideas to the finalized manuscript. YB designed experiments and contributed to manuscript revision. HD designed experiments, analyzed data, and contributed to manuscript drafting. BY supervised all the experiments and contributed to manuscript revision. All authors contributed to the article and approved the submitted version.

## Funding

This study was supported by National Natural Science Foundation of China (grant no. 81802182), Guangdong Medical Science Research Foundation (grant no. B2019040), Science and technology program of Guangdong Province (grant no. 2016B090913004), the Innovation Leader Team Program of Guangzhou (grant no.201809010014), President Foundation of Nanfang Hospital, Southern Medical University (grant no. 2020C027), Postdoctoral Science Foundation of China (grant no. 2021M701635) and Natural Science Foundation of Guangdong Province (grant no. 2019A1515012115).

## Conflict of Interest

The authors declare that the research was conducted in the absence of any commercial or financial relationships that could be construed as a potential conflict of interest.

## Publisher’s Note

All claims expressed in this article are solely those of the authors and do not necessarily represent those of their affiliated organizations, or those of the publisher, the editors and the reviewers. Any product that may be evaluated in this article, or claim that may be made by its manufacturer, is not guaranteed or endorsed by the publisher.
