# Supplementary material for: Association of Antibiotic Alterations in Gut Microbiota With Decreased Osseointegration of an Intramedullary Nail in Mice With and Without Osteomyelitis
Source: Front Endocrinol (Lausanne). 2021 Dec 9;12:774257. doi: 10.3389/fendo.2021.774257 (PMC8696274; doi:10.3389/fendo.2021.774257)
Supplement: Supplementary file 1 [file DataSheet_1.docx]

**Association of antibiotic alterations in gut microbiota with decreased osseointegration of an intramedullary nail in mice with and without osteomyelitis**

Xingqi Zhao^1†^, Zhaohui Zhang^2, 3, 4†^, Yiran Wang^1, 2^, Kai Qian^3^, Hanjun Qin^1^, Haoyang Wan^1^, Shihao Wang^3^, Zhengwen Zhu^3^, Siqi Yang^3^, Nan Jiang^1^, Yifang Zhang ^5^, Yang Bai^3*^, Huimin Deng^2, 3 *^, Bin Yu^1*^

^1^ Department of Orthopedics, Nanfang Hospital, Southern Medical University, Guangzhou, China & Guangdong Provincial Key Laboratory of Bone and Cartilage Regenerative Medicine, Nanfang Hospital, Southern Medical University, Guangzhou, China.

^2^ Department of Gastroenterology, Huizhou Municipal Central Hospital, Huizhou, China.

^3^ Guangdong Provincial Key Laboratory of Gastroenterology, Institute of Gastroenterology of Guangdong Province & Department of Gastroenterology, Nanfang Hospital, Southern Medical University, Guangzhou, China.

^4^ Department of Gastroenterology, The First Affiliated Hospital of Jinan University, Jinan University, Guangzhou, China.

^5^ Editorial office, Chinese Journal of Orthpopaedic Trauma, Nanfang Hospital, Southern Medical University, Guangzhou, China.

**Supplementary materials**

S. Fig 1. **Gut microbiota dysregulation induced by antibiotics showed no significant effects on liver and kidney.** Liver and kidney specimens were harvested at 4 weeks postsurgery from mice in untreated, antibiotic-treated (Ab), chronic osteomyelitis (CO), antibiotic-treated CO (Ab-CO), implant (Im), and antibiotic-treated implant (Ab-Im) groups. **a.** H&E staining of liver (200× magnification). **b.** H&E staining of kidney (200× magnification). **c.** Serum level of alanine transaminase (ALT) in all groups. **d.** Serum level of aspartate aminotransferase (AST) in all groups. **e.** Serum level of blood urea nitrogen (BUN) in all groups. **f.** Serum level of creatinine in the all groups. Data for liver and kidney function indexes are shown as median and interquartile range. All data were analyzed by unpaired *t*-test between untreated and Ab, Ab-CO and CO, or Ab-Im and Im groups.

S. Fig 2. **Gut microbiota dysregulation induced by antibiotics had negative impact on intestinal mucosa and glandular structure of colon.** Colon specimens were harvested at 4 weeks postsurgery from mice in untreated, antibiotic-treated (Ab), chronic osteomyelitis (CO), antibiotic-treated CO (Ab-CO), implant (Im), and antibiotic-treated implant (Ab-Im) groups. H&E staining of colon (200× magnification) in all groups.

S. Fig 3. **A high dose broad-spectrum antibiotic cocktail significantly disrupted host gut microbiota.** After 5 weeks of antibiotic treatment, intestinal contents specimens were harvested and analyzed by 16S rRNA sequencing from mice (*n*=5–6 mice/group): the untreated group, the chronic osteomyelitis (CO) group, the antibiotic-treated CO (Ab-CO) group, the implant (Im) group, and the antibiotic-treated implant (Ab-Im) group. **a.** Shanon index of all groups; **b.** Relative abundance of *Firmicutes* phylum; **c.** OTU number of *Bifidobacterium* genus, **d.** Principal co-ordinate analysis (PCoA) using unweighted UniFrac distances based on OTUs; **e.** Different genus observed among all groups based on metastats analysis. **f.** Relative abundance of top 10 classes in all groups. Data for Shanon index, OTU number and relative abundance are shown as median and interquartile range. All data were analyzed by unpaired *t*-test between Ab-CO and CO, or between Ab-Im and Im groups.
