# Supplementary figures and images for: Association of Antibiotic Alterations in Gut Microbiota With Decreased Osseointegration of an Intramedullary Nail in Mice With and Without Osteomyelitis
Source: Front Endocrinol (Lausanne). 2021 Dec 9;12:774257. doi: 10.3389/fendo.2021.774257 (PMC8696274; doi:10.3389/fendo.2021.774257)

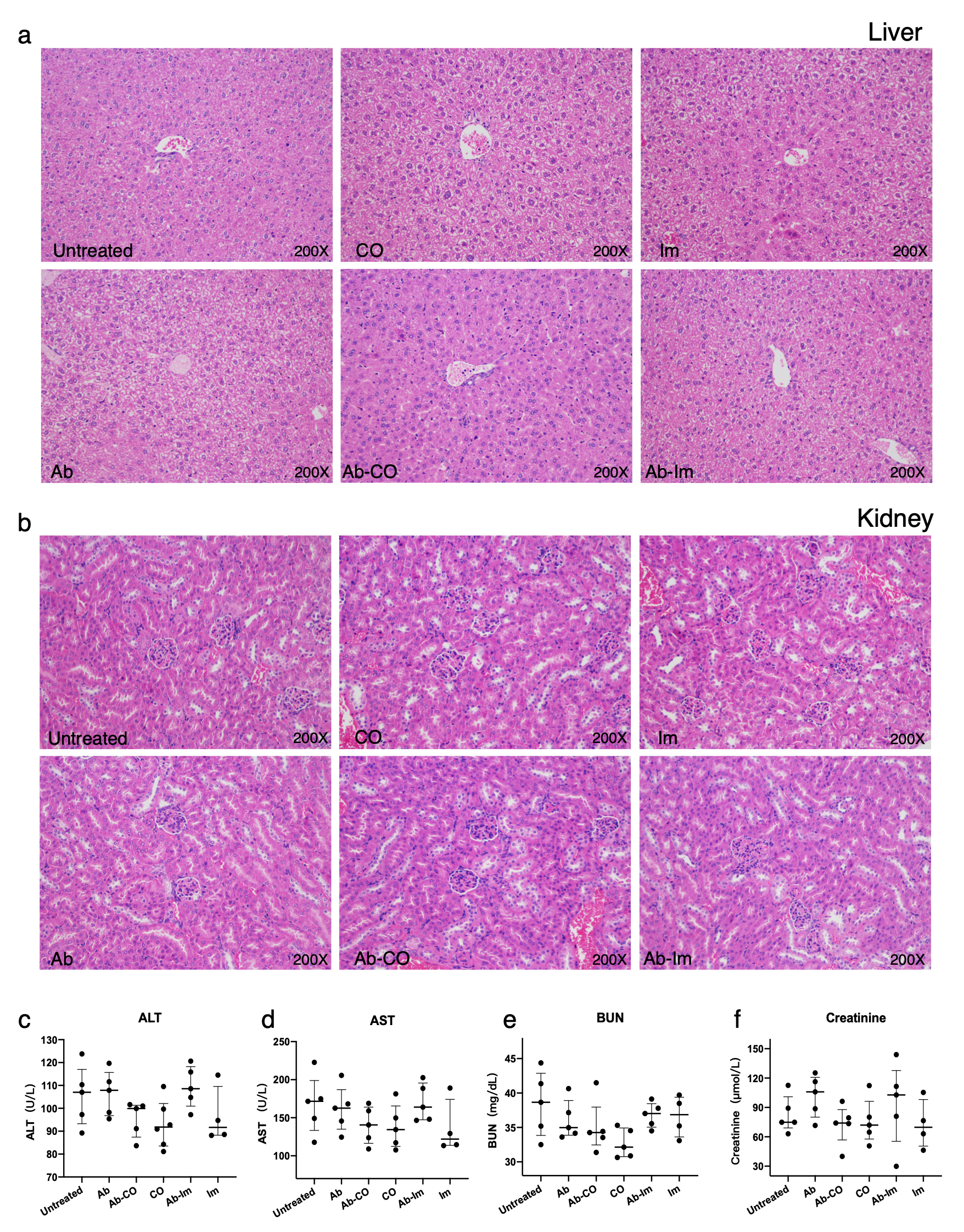

Supplement: Supplementary file 2 [file Image_1.jpeg]

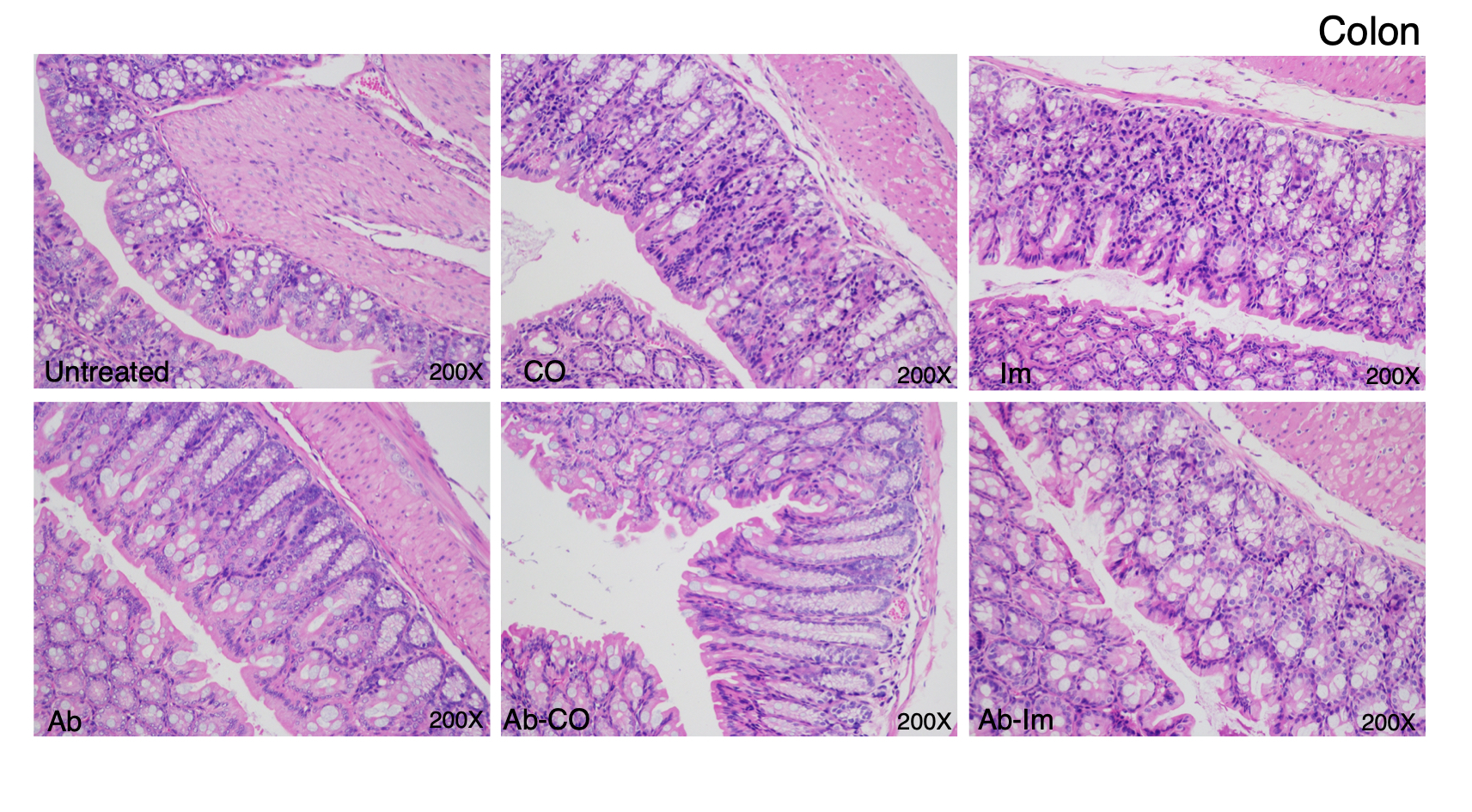

Supplement: Supplementary file 3 [file Image_2.jpeg]

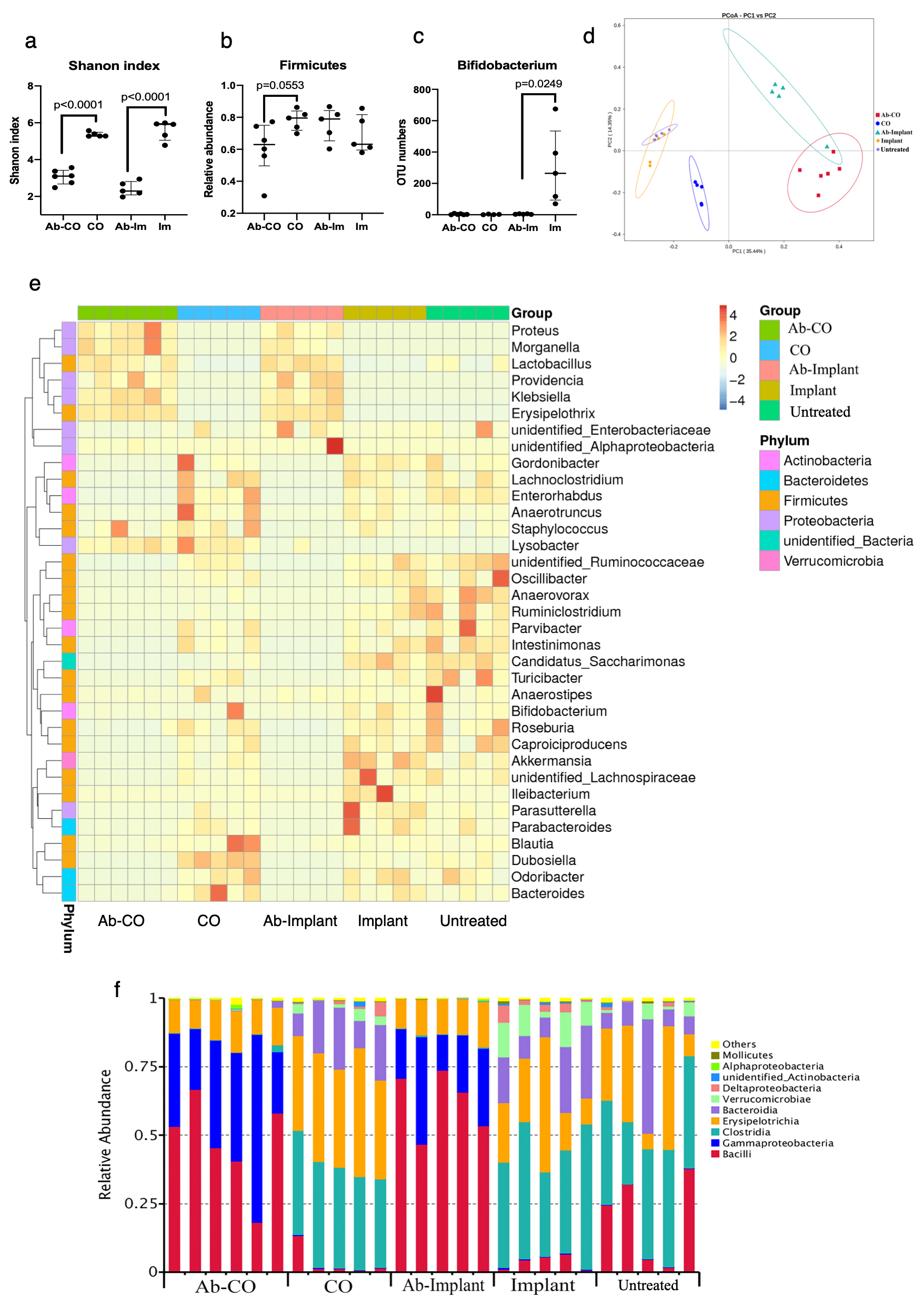

Supplement: Supplementary file 4 [file Image_3.jpeg]
